# Supplementary material for: Chemoradiotherapy Combined with Brachytherapy for the Definitive Treatment of Esophageal Carcinoma
Source: Cancers (Basel). 2023 Jul 12;15(14):3594. doi: 10.3390/cancers15143594 (PMC10377190; doi:10.3390/cancers15143594)
Supplement: Supplementary file 1 [file cancers-15-03594-s001.zip › cancers-2418391-supplementary.pdf]

cancers MDPI

Table S1. S: Comparison of acute toxicity in patients receiving BT or EBRT boost versus BT and EBRT boost.

Acute Toxicity Grade	BT or EBRT Boost		BT and EBRT Boost	
	N Patients	Rate (%)	N Patients	Rate (%)
0	2	2.4%	2	3.2%
1	22	25.9%	27	42.9%
2	46	54.1%	24	38.1%
3	13	15.3%	9	14.3%
4	1	1.2%	0	0.0%
5	1	1.2%	1	1.6%

Table S2. Comparison of late toxicity in patients receiving BT or EBRT boost versus BT and EBRT boost.

Late Toxicity Grade	BT or EBRT Boost		BT and EBRT Boost	
	N Patients	Rate (%)	N Patients	Rate (%)
0	61	71.8%	37	58.7%
1	7	8.2%	8	12.7%
2	5	5.9%	4	6.3%
3	9	10.6%	10	15.9%
4	1	1.2%	1	1.6%
5	2	2.4%	3	4.8%

Table S3. Odds ratios (OR) for receiving brachytherapy before and after propensity score matching (PSM).

	Before PSM (N = 183)				After PSM (N = 94)			
	OR	95% CI		*p*	OR	95% CI		*p*
		Lower	Upper			Lower	Upper	
age	0.99	0.96	1.02	0.325	0.97	0.93	1.01	0.161
UICC stage	0.63	0.46	0.85	0.003	0.90	0.59	1.35	0.600
KPS	1.05	0.84	1.31	0.697	1.01	0.75	1.36	0.940
grade	1.16	0.67	2.04	0.596	1.31	0.60	2.82	0.498
location	1.48	1.01	2.16	0.043	1.12	0.66	1.90	0.685
histology	0.87	0.45	1.67	0.679	0.92	0.41	2.07	0.836

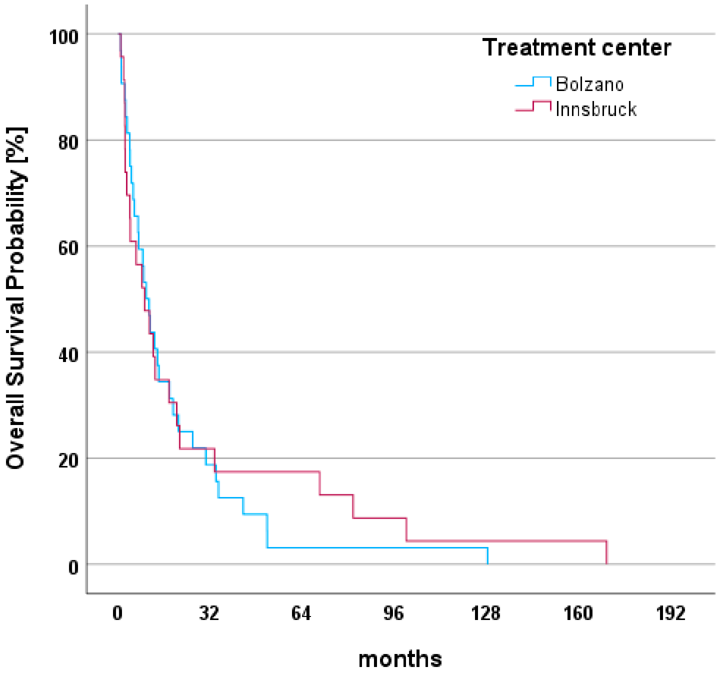

Figure S1. Kaplan Meier plots of center specific survival. Analysis of patients with comparable characteristics and treatment not receiving brachytherapy.

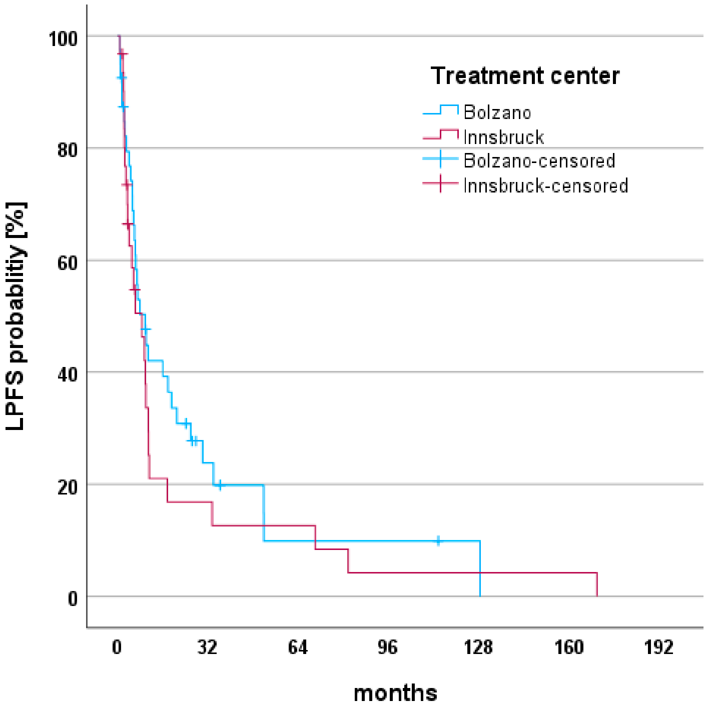

Figure S2. Kaplan Meier plots of center specific LPFS. Analysis of patients with comparable characteristics and treatment not receiving brachytherapy.
